# Systems biology for reverse aging

**DOI:** 10.18632/aging.203188

**Published:** 2021-06-09

**Authors:** Kwang-Hyun Cho, Sugyun An, Junsoo Kang

**Affiliations:** 1 Department of Bio and Brain Engineering, Korea Advanced Institute of Science and Technology (KAIST), Daejeon, 34141, Republic of Korea

**Keywords:** systems biology, cellular senescence, geroconversion, network modeling, PDK1, skin aging

Cellular senescence is an irreversible and permanent cell cycle arrest in response to internal and external stresses. Its unresponsiveness to growth factor signals distinguishes it from a potentially reversible state, quiescence. Cellular senescence can inhibit tumor development by blocking proliferation of damaged cells, but as senescent cells become accumulated in a tissue, they can contribute to the promotion of age-related diseases such as cancer by secreting inflammatory cytokines [1]. To tackle this problem, various therapeutic strategies including clearance of senescent cells, suppression of harmful cytokines, and partial reprogramming through temporary expression of Yamanaka factors (Oct4, Sox2, Klf4, c-Myc) are being actively investigated [1,2]. However, those therapeutic strategies have some critical drawbacks. For instance, clearance of senescent cells can interfere with tissue regeneration since senescent cells help recruit immune cells to the wound site and provide proliferation signals during tissue repair. Suppression of inflammatory cytokines may also interfere with immune surveillance on cancer cells and other pathogens. As for partial reprogramming, although partial reprogramming proved that senescent cells can be reverted, early termination of this reprogramming process is known to cause epigenetic dysregulation, resulting in dedifferentiated dysplastic cells such as renal cancer [3]. Therefore, a novel therapeutic strategy without such critical limitations is highly needed.

Cellular senescence is caused by complex interactions among biomolecules that govern cell cycle, DNA damage response, energy metabolism and cytokine secretion [4]. Recent studies showed that cellular senescence, previously considered as an irreversible biological phenomenon, can be reversed, but due to the nature of such complex interactions governing cellular senescence, the mechanism by which cellular senescence can be reversed has not been revealed [2,5]. Moreover, when senescent cells are reverted without sufficient mechanistic understanding, those reverted cells can contribute to the development of uncontrolled proliferation, leading to cancer [3,5]. To identify protein targets capable of reversing cellular senescence without inducing cancer and to elucidate the underlying mechanism, An et al. [4] conducted a systems biology study by comprehensively analyzing the complex regulatory interactions involved in senescence.

**Figure 1:**
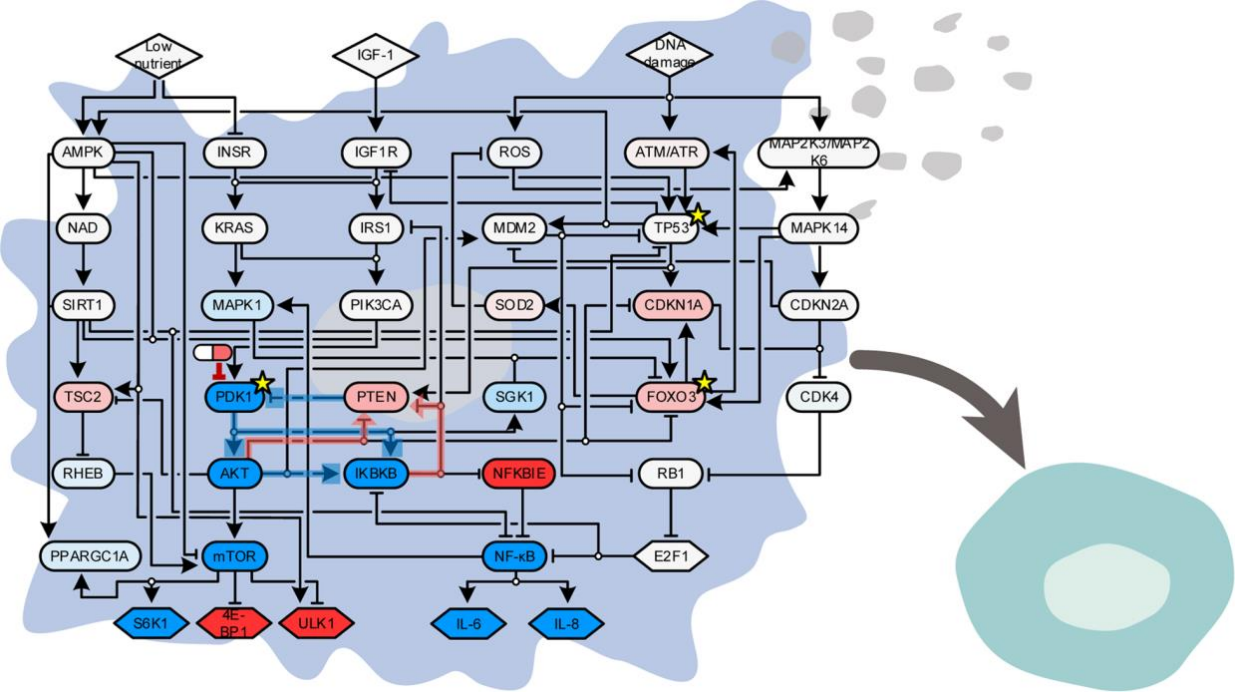
**Systems biology identifies a promising target for reverse aging in human dermal fibroblasts.** Inhibition of PDK1 reverts senescent cells (left) to quiescent cells (right) without inducing uncontrolled proliferation in human dermal fibroblasts. Nodes are colored according to whether PDK1 inhibition from cellular senescence attractor state increases (red) or decreases (blue) the node activity, and edges that constitute the core positive feedback loop are colored depending on their signs of signal flows; positive (red) or negative (blue) (see An et al. [4] for more details). Three nodes that constitute minimal feedback vertex set are marked by yellow stars.

An et al. reconstructed an ensemble of 5000 Boolean network models that can represent senescence, quiescence, and proliferation phenotypes by integrating information from the literature, network databases and phosphoprotein array data of dermal fibroblasts they measured [4]. In their models, cellular senescence is induced by simultaneous activation of DNA damage signal (doxorubicin) and growth signal (IGF-1 plus serum), which coincides with the geroconversion theory [4,6]. They identified 3-phosphoinositide-dependent protein kinase 1 (PDK1) as the optimal protein target that can safely revert senescence to quiescence while avoiding uncontrolled proliferation, through extensive computer simulation analysis of the ensemble model. Furthermore, by applying network control theory [7], they ascertained that PDK1 constitutes the minimal feedback vertex set (Figure 1) that plays a crucial role in determining steady-state network dynamics and forms a positive feedback structure along with AKT, IKBKB and PTEN, that simultaneously control both nuclear factor κB, which controls cytokine secretion, and mTOR, which regulates cell growth (Figure 1).

In order to validate the simulation results, they conducted *in vitro* experiments and confirmed that when PDK1 was inhibited, various markers of cellular senescence are returned to normal and proliferation potential is restored [4]. From wound healing assays and 3D reconstructed skin tissue experiments, they also reaffirmed that the reverted cells are able to respond appropriately to external stimuli. In particular, by observing dermal fibroblast within dermis along with keratinocyte within epidermis, 3D reconstructed skin tissue experiments verified that PDK1 inhibition promotes epidermal renewal and restores skin thickness, resulting in reversal of age-related skin degeneration.

An et al. [4] proved that PDK1 inhibition can safely revert senescent cells without the risk of causing tumorigenesis using systems biology approach. Since PDK1 is known as an oncogene, the result seems to be more promising. This study provides a novel insight into the complex regulatory mechanism of cellular senescence and suggests a potential therapeutic strategy for treating age-related diseases that are associated with the accumulation of senescent cells. Moreover, by extending the reconstructed network to comprehensively represent cellular senescence for multiple tissues and stimuli, synergistic combination protein targets of PDK1 may also be identified.

The study also provides an insight into cancer therapy. Cancer and cellular senescence have a close relationship. A senescent cell inhibits its own tumorigenesis but at the same time promotes tumorigenesis of surrounding cells by secreting harmful cytokines. Furthermore, when cancer cells are subjected to a high level of DNA damage during drug treatment, they may turn into senescent cells. These cancer-derived senescent cells tend to cause drug resistance or even recurrence of more aggressive cancer. Considering this, a two-step therapeutic strategy that induces cancer cells into senescent cells and then removes the senescent cells was proposed [5]. Based on the findings from An et al., a different two-step treatment that reverts cancer-derived senescent cells to normal cells can be considered. In particular, in case of cancer reversion, Lee at al. showed its feasibility by identifying SETDB1, a key regulator that can revert colon cancer cells into normal colon cells, using systems biology approach [8].
